# Probabilistic 3D lithology classification from elastic property volumes an advanced inversion workflow at Desouq Gas Field, West Nile Delta, Egypt

**DOI:** 10.1038/s41598-026-42888-z

**Published:** 2026-03-27

**Authors:** Mohamed Said El Hateel, Abdel Moktader A. El Sayed, Abdel-Khalek El-Werr, Ahmed Abdel-Hady

**Affiliations:** 1 Suez Oil Company (Suco), Cairo, Egypt; 2 Geophysics Department, Faculty of Science, Ain Shams University, Cairo, Egypt; 3 Harbour Energy Plc. Egypt Branch , Cairo, Egypt

**Keywords:** Rock Physics Modeling, Seismic Elastic Inversion, PDFs, Lithology Probability Cubes, Energy science and technology, Solid Earth sciences

## Abstract

Advanced 3D lithology prediction is vital for reducing uncertainty in reservoir characterization and exploration planning. Traditional post-stack inversion yielded only acoustic impedance volumes, limiting facies discrimination. However, pre-stack simultaneous inversion enables direct estimation of elastic property volumes, particularly P-impedance, shear impedance and Vp/Vs, linking seismic inversion to rock physics evaluation. In this study, the applied workflow integrates seismic inversion products and borehole information within a structured, multi-stage lithology classification framework. Initially, 3D seismic inversion volumes and available well logs were subjected to comprehensive quality control and jointly interpreted to establish a consistent geological framework. This geological interpretation guided subsequent petrophysical analysis of the well logs, from which key reservoir properties were derived. Based on these petrophysical results, rock physics crossplots were conducted to define and discriminate the lithology classes generating a litho-facies log for each well and characterize their elastic responses. The classified well-based data were then, used to generate probability density functions (PDFs) for each lithology class, forming the statistical foundation of the lithology classification model. The dataset used to train an ML algorithm (trained model) was subsequently applied to the 3D seismic inversion volumes to predict lithological distributions away from the wells. Finally, the resulting lithology classification volumes were visualized, interpreted, and quality-controlled to delineate reservoir outlines and assess their spatial continuity and geological credibility. This workflow applied to the Abu Madi Formation in the west onshore Nile Delta, with a focus on the Desouq Gas Field. The probabilistic classification revealed compartmentalized gas-sand channels, refined hydrocarbon facies outlines in the northwest sector, and identified eight previously unrecognized gas-charged zones in the southwest sector. Validation using classification metrics and confusion-matrix analysis confirmed the robustness of the workflow, while integration with elastic property crossplots clarified ambiguities caused by thin anhydrite layers that commonly generate misleading amplitude responses which reduced misclassification risks. The resulting 3D lithology volumes (gas sand, wet sand, shale, and tight anhydrite formation) provide enhanced insights into subsurface heterogeneity and hydrocarbon potential, demonstrating the added value of integrating seismic inversion, machine learning, and rock physics analysis.

## Introduction

Desouq Gas Field study area is a part of West onshore Nile Delta province, located within the central-western part of the onshore Nile Delta, southeast of the offshore Abu Qir Gas Field. Its lease covers approximately area of 365 km² and supported with well-established infrastructure that facilitate regional oil and gas production and transportation. The central point of study area lies about 79 km east southeast of Alexandria and 104 km southwest of Damietta, with the western boundary closed to Rosetta branch of the Nile River.

Despite extensive exploration in the Nile Delta, hydrocarbon prediction in this area remains challenging due to complex depositional environments, structural heterogeneity, and seismic imaging limitations. Key uncertainties include the risk of encountering non-productive or low-quality sandstone intervals, difficulties in defining the lateral and vertical extent of gas-bearing reservoirs, and distinguishing true gas-related seismic amplitude anomalies from false responses generated by high-impedance anhydrite layers. These challenges necessitate integrated workflows that combine seismic inversion, rock physics, and probabilistic classification.

Probabilistic 3D lithology classification has been successfully applied in several areas worldwide^1–3^ demonstrating its adaptability to complex depositional settings. However, its application in the onshore Nile Delta remains limited, where previous studies^4–6^ have focused primarily on deterministic facies mapping, amplitude-based interpretation, or traditional seismic inversion.

These earlier approaches relied on post-stack inversion that produced only acoustic impedance volumes, restricting facies discrimination and failing to resolve ambiguities caused by thin anhydrite layers and compartmentalized sandstone reservoirs. In contrast, pre-stack simultaneous inversion enables direct estimation of elastic property volumes (P-impedance, shear impedance, and Vp/Vs ratio), which, when integrated with probabilistic classification and well-log calibration, generate lithology probability cubes that better capture subsurface heterogeneity and fluid variability.

This study addresses that gap, by applying the 3D probabilistic lithology prediction workflow to the Abu Madi Formation in the Desouq Gas Field, by integrating well logs, amplitude-versus-offset (AVO) analysis, pre-stack inversion results, and rock physics modeling.

The aim of this study is to build a 3D probabilistic lithology classification volume considering reservoir properties that can highlight and quantify the pay as hydrocarbon pore volume versus the non-productive sections. The lithology classification is integrated by two processes^7^.

1) Lithology analysis process to generate a litho-classification model, which is the frequency distribution of the samples relevant to each class create probability density functions (PDFs) from the cluster analysis of the log data. These PDFs represent the variability in the formation properties. The log properties are up-scaled to seismic properties, and the PDFs are aligned to the expected seismic inversion accuracy.

2) Lithology prediction process, where the probability density functions (PDFs) are applied to the elastic attribute cubes (P-impedance, shear impedance, and Vp/Vs ratio) from the pre-stack seismic inversion to produce lithology and fluid prediction volumes with their associated uncertainties.

These two processes have been carried out for probabilities using Petrel™, version 2020 software, quantitative interpretation (QI) module, developed by Schlumberger.

This approach refines facies outlines, reduces misclassification risks, and enhances reservoir mapping, thereby advancing reservoir characterization in the Nile Delta and providing a transferable methodology for comparable global settings.

## Geologic setting

The Nile Delta is structurally divided into several tectono-sedimentary provinces that control hydrocarbon distribution. Early studies^8^ highlighted the importance of a regional hinge zone separating different structural domains. Later work^9–11^ confirmed the contrast between a southern uplifted block and a northern subsiding basin. Building on these studies, the Nile Delta subdivided into three main provinces for hydrocarbon exploration^12^: the South Nile Delta Block, the North Nile Delta Basin, and the deep offshore province. The southern block is relatively stable with thinner sediments, whereas the northern basin contains thicker sedimentary deposits and more active faulting. The deep offshore represents the northern continuation of this basin into the Mediterranean, where significant gas accumulations occur due to high sedimentation and subsidence rates. Moreover, integration of seismic interpretation, quantitative petrophysical analysis, and 3D geological modeling, enable detailed spatial characterization of reservoir facies distribution and heterogeneity^13^.

**Fig. 1:**
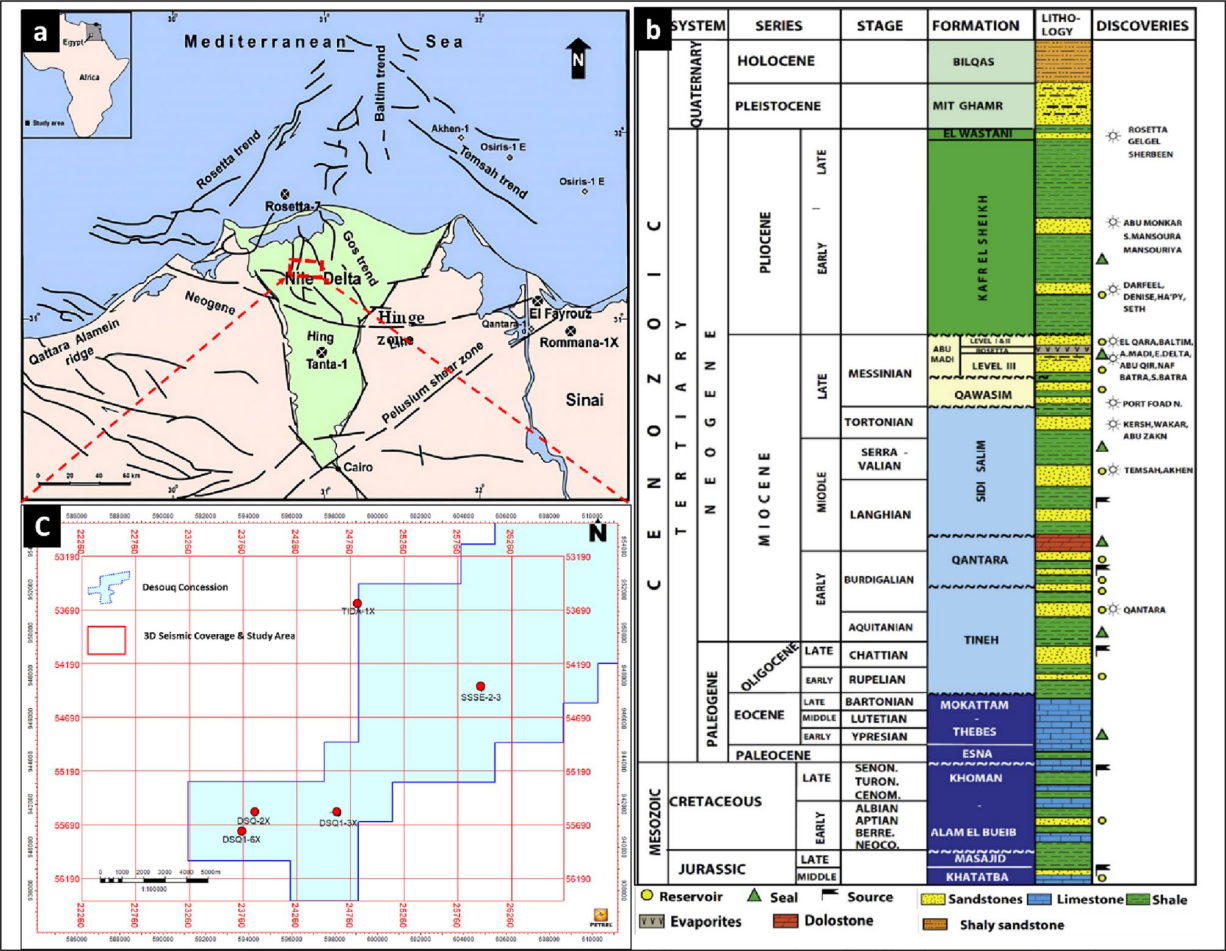
Nile Delta general tectonic regime, after^11^ (**a**), The generalized stratigraphic column of the Nile Delta, after^18^ (**b**), 2D map view shows, well locations, seismic coverage and Desouq study area generated by Petrel™ software (version 2020) developed by Schlumberger (**c**).

The onshore Nile Delta exhibits a complex tectonic framework shaped by successive phases of rifting, subsidence, and inversion (Fig. 1a). Its present architecture reflects Mesozoic extensional activity associated with the opening of the Neo-Tethys Ocean, which generated major normal fault systems. Among these systems are the NE-SW-oriented Rosetta Fault System, the E-W aligned Nile Delta Hinge Zone, and the NW-SE-oriented Temsah Fault System^11,14–16^. These structures controlled basin subsidence, influenced sediment dispersal, and played a key role in reservoir compartmentalization and hydrocarbon migration pathways.

The Disouq Concession is located in the central part of northern Egypt (Fig. 1c), close to the Mediterranean Sea. It forms a part of the onshore Nile Delta, extending from the town of Tanta in the south to Lake Burullus in the north, and is traversed in the west by the Rosetta branch of the Nile River. The concession straddles two tectono-stratigraphic provinces separated by the Nile Delta Hinge Zone (NDHZ), which trends NW-SE and rotates southwestward. The central Disouq Segment of the NDHZ is interpreted as the western boundary of a failed rift margin extending southwestward toward the Gulf of Suez Clysmic fault system^17^. The interaction between the hinge zone and adjacent fault systems created accommodation space and localized depocenters, strongly influencing sediment thickness, facies distribution, and reservoir quality.

Stratigraphically, the Nile Delta records a thick succession of fluvial, deltaic, and marine deposits ranging from the Mesozoic to the Quaternary^16,18^. Reservoir intervals are primarily Oligocene to Late Pliocene in age (Fig. 1b). Three major depositional cycles characterize the clastic fill:

- **Miocene cycle**: Represented by the Sidi Salim, Qawasim, and Abu Madi formations, comprising non-marine to shallow marine deposits. A major sea-level fall during the Messinian Salinity Crisis led to Mediterranean drawdown, canyon incision, and deposition of thick evaporates^19^.
- **Plio-Pleistocene cycle**: Includes the open-marine Kafr El Sheikh Formation and the deltaic El Wastani, Mit Ghamr, and Bilqas formations, reflecting renewed marine transgression and progradation.
- **Holocene cycle**: Encompasses the uppermost stratigraphy, dominated by fluvial and deltaic deposits that continue to shape the modern delta plain.

Overall, the interplay between tectonic structures (Rosetta Fault, Temsah Fault, and NDHZ) and sedimentary processes has produced a highly heterogeneous stratigraphic framework. Fault-controlled subsidence created accommodation space, while hinge zone rotation influenced sediment pathways and reservoir distribution. This structural-sedimentary coupling is critical for understanding reservoir architecture and hydrocarbon prospectivity in the Nile Delta^15,16,18^.

## Materials and methods

The available materials in the study area are a combination of seismic and well-logging information, which are critical for reservoir characterization and lithology identification. The seismic set includes a 3D Full-stack seismic cube, partial angle stacks (near offset measurements of 0°−15°, middle offset measurements of 15°−30°, and far offset measurements of 30°−45°), and well velocity surveys for depth measurement. Along with five wells provide information for well logging, which comprises a full sequence of parameters such as Gamma Ray, Density, Sonic measurements of both compression and shear waves, Neutron, Resistivity, and Spontaneous Potential.

**Fig. 2:**
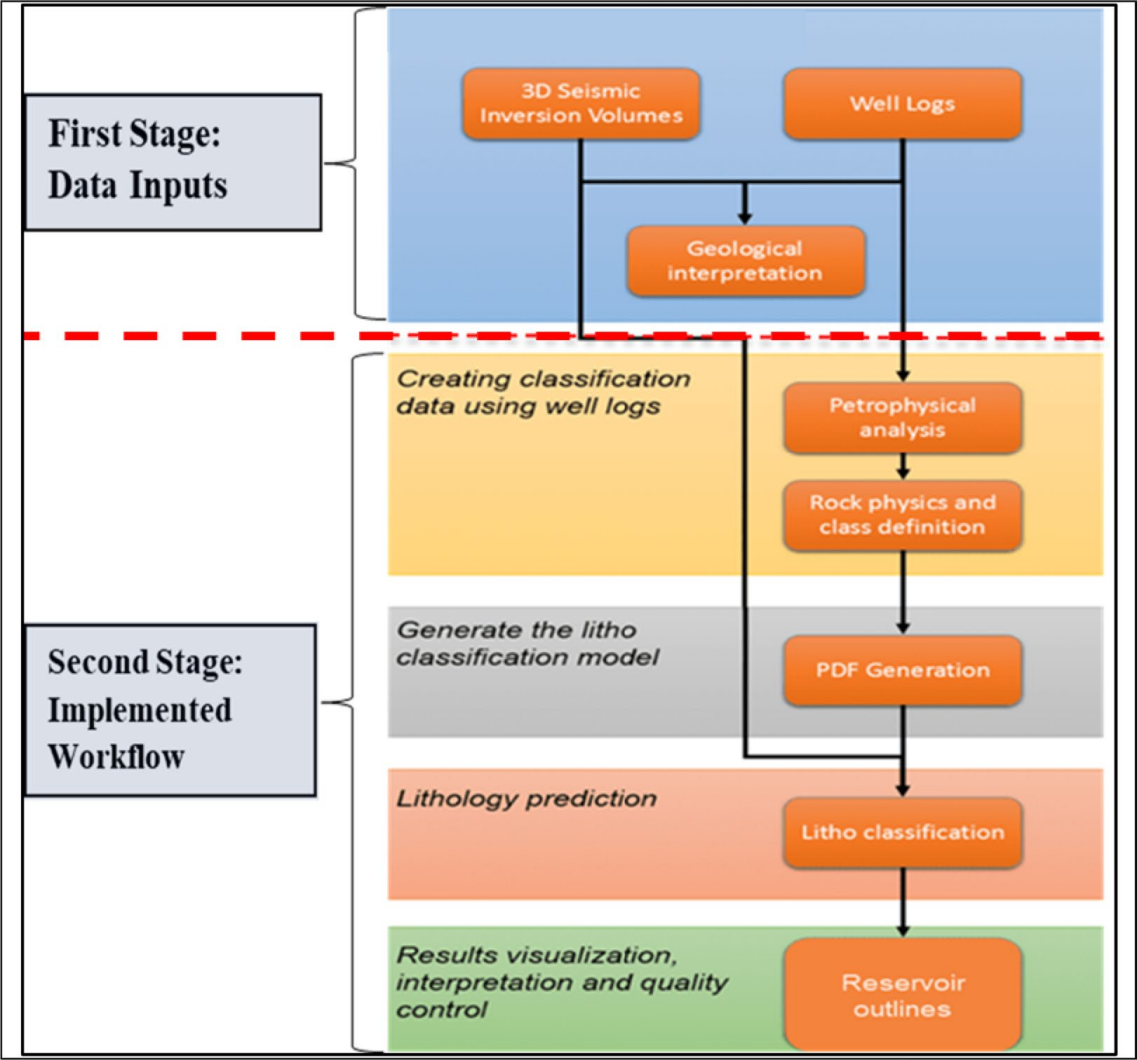
Summarizes the full sequence steps required to execute the lithology classification and prediction workflow, after^7^.

Before implementing the lithology classification and prediction methodology, a comprehensive preparatory study was undertaken. The workflow includes two stages^7^: (a) **The first stage** is **Data Inputs** and their quality control, which include well logs, geological and seismic interpretation, and 3D seismic elastic inversion volumes, (b) **The second stage** is the **Implemented Workfl**ow, which includes: Petrophysical analysis, rock physics modeling and class definition, property distribution function (PDF) generation, litho classification volumes prediction, and finally reservoir outlines as shown in Fig. 2.

### Data inputs: Stage 1

As illustrated in Fig. 2, the process begins with input data conditioning and its quality control, including verification of well-log datasets, geological and seismic interpretation and 3D seismic elastic inversion volumes as follows:

### Well-logs

Well-log datasets provide the foundation for a systematic lithology-classification workflow that includes several key stages: visualization and data analysis, adjustment and validation of formation tops, and assessment of facies spatial distribution in relation to structural setting.

**Fig. 3:**
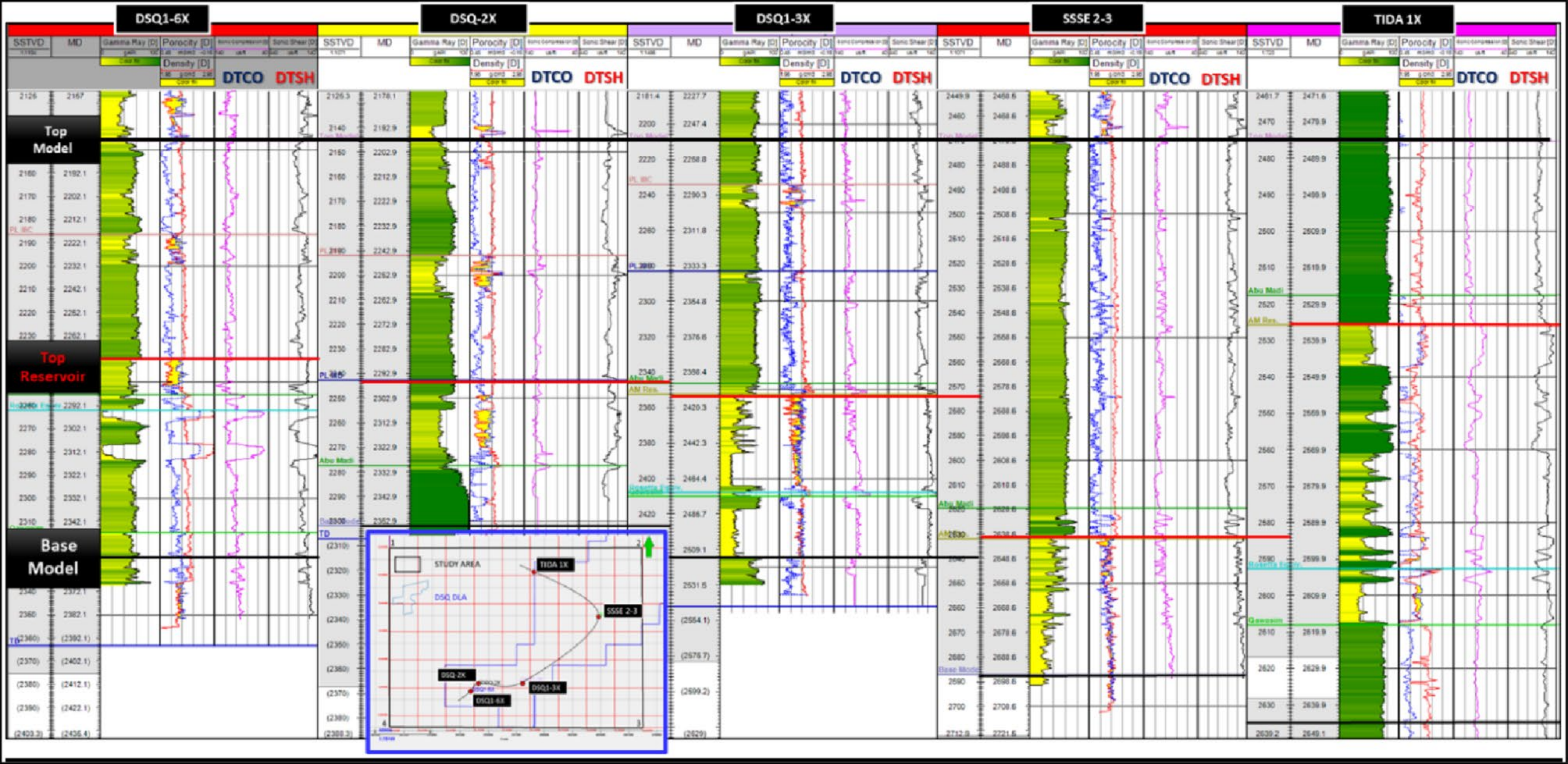
Well logs correlation panel of the DSQ 1 6X, DSQ 2X, DSQ1 3X, SSSE 2 3, and TIDA 1X wells.

Figure 3 presents the well-log correlation panel for the DSQ-1-6X, DSQ-2X, DSQ-3X, SSSE-2–3, and TIDA-1X wells.

### Seismic interpretation

Seismic interpretation in the study area was conducted using 3D seismic data by Petrel™ software (version 2020) developed by Schlumberger. The interpretation focused on identifying three key geologic horizon tops and their associated fault systems, which were tied to the studied wells through well-to-seismic correlation.

**Fig. 4:**
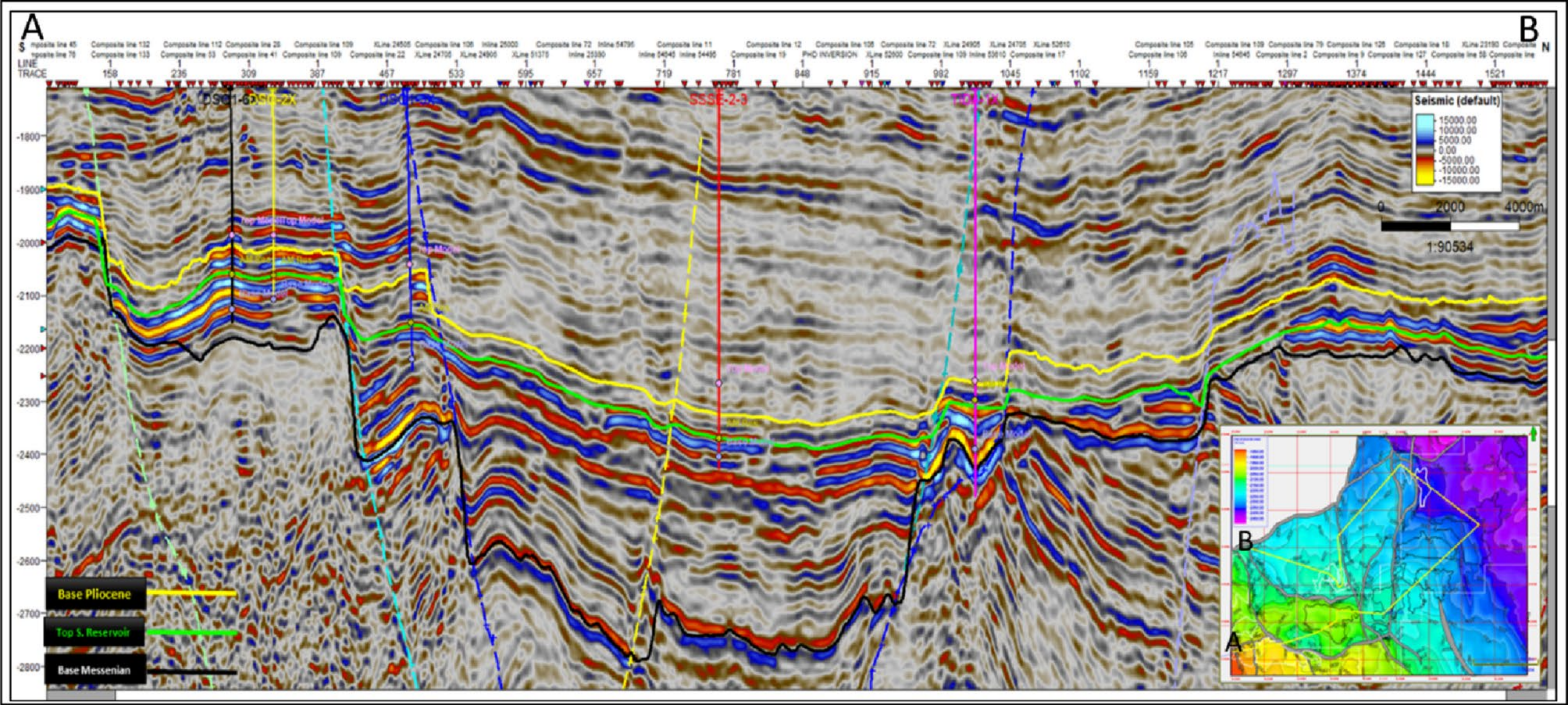
Interpreted arbitrary seismic line passing through the studied wells.

These horizon tops, arranged stratigraphically from young to old as follows: the top of Abu Madi Formation (Base Pliocene) represented by yellow color in the interpreted arbitrary seismic section shown in Fig. 4, the top of Abu Madi sand reservoir (green color) and its involved time structure map shown in the right bottom corner of the seismic section, and Qawasim Formation (Base Messenian; blue color). The interpreted seismic sections revealed a network of structural elements dissecting these horizons. Compartmentalized gas-sand channels are clearly observed within the Abu Madi reservoir.

Figure 4 presents an arbitrary seismic line passing through DSQ-1-6X, DSQ-2X, DSQ-3X, SSSE-2–3, and TIDA-1X wells. It illustrates the interpreted geologic horizon tops and their dissecting structural framework. This interpretation provides the structural and stratigraphic foundations for subsequent seismic inversion, elastic property modeling, and lithology prediction workflow.

### Simultaneous pre-stack seismic inversion

Seismic inversion is a quantitative technique used to derive subsurface earth models from seismic reflection data. Simultaneous pre-stack seismic elastic inversion in particular enables the joint estimation of key elastic parameters, most notably acoustic impedance and shear impedance, from angle-dependent seismic amplitudes. Recent advancements have extended its application toward direct prediction of lithology and fluid types, including gas-bearing sands, wet sands, anhydrite (tight formations), and shale content^20,21^.

Pre-stack inversion transforms pre-stack seismic gathers into petrophysical properties such as acoustic impedance, shear impedance, lithology, and porosity through the application of inversion algorithms constrained by geological and petrophysical inputs. When integrated with well-log data, rock-physics analysis, and geological interpretation, pre-stack seismic inversion provides an enhanced understanding of reservoir architecture, fluid distribution, and production potential, an approach that has proven particularly valuable in the Nile Delta^22^.

In this study, simultaneous pre-stack inversion was applied to generate elastic property volumes, specifically P-impedance (PI), shear impedance and Vp/Vs ratio. These seismic properties are used primarily as indicators in lithology classification and in the identification of gas-charged channel systems. In gas-bearing reservoirs, significant contrasts are observed in P-impedance, Vp/Vs ratio, and density. This contrast facilities distinguishing gas-bearing sands within the Abu-Madi Formation of the western Nile Delta, and particularly in the Desouq Gas Field study area.

**Fig. 5:**
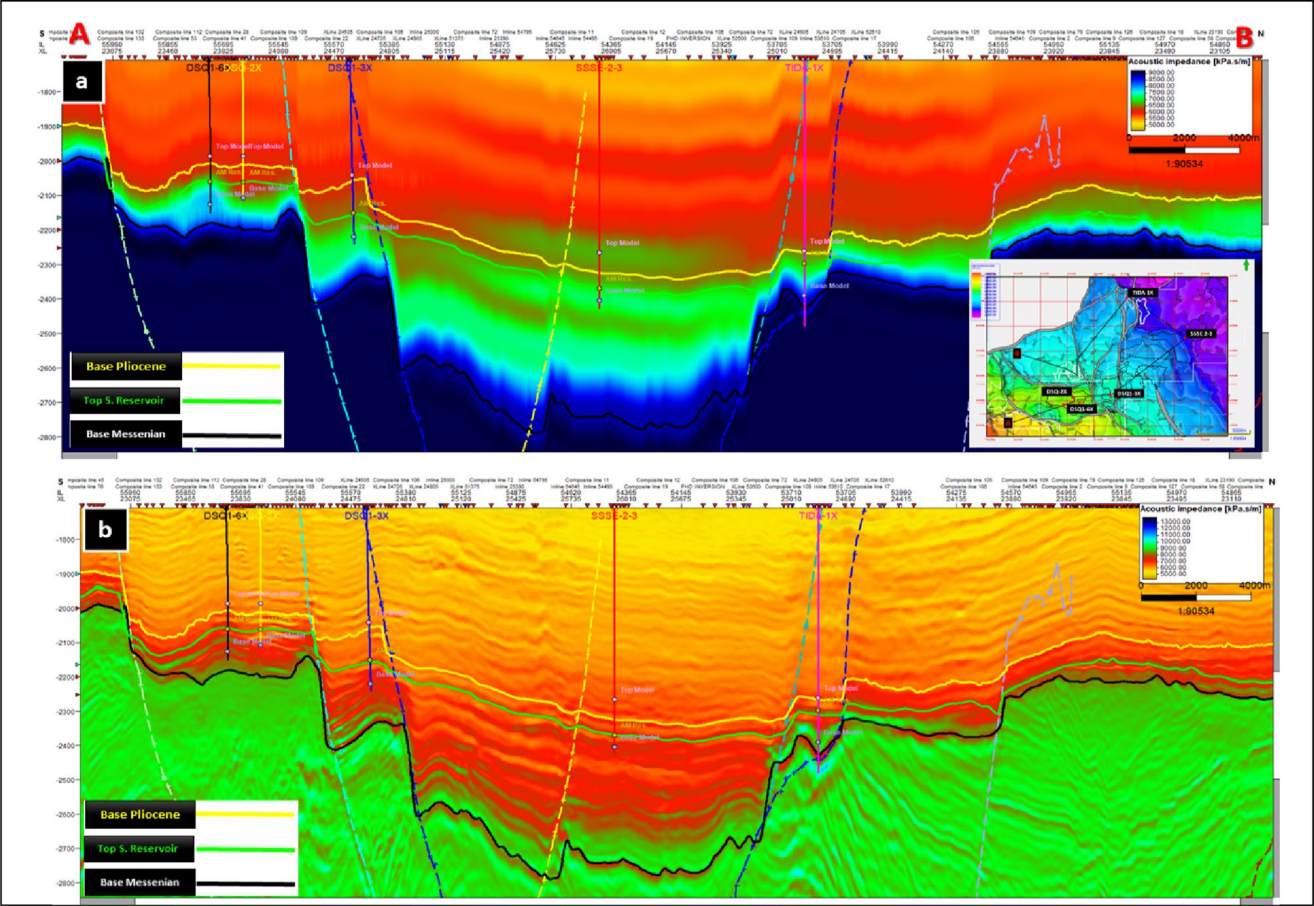
LFM (**a**) and Inverted P-impedance (**b**) along the arbitrary line passing through the studied wells.

**Fig. 6:**
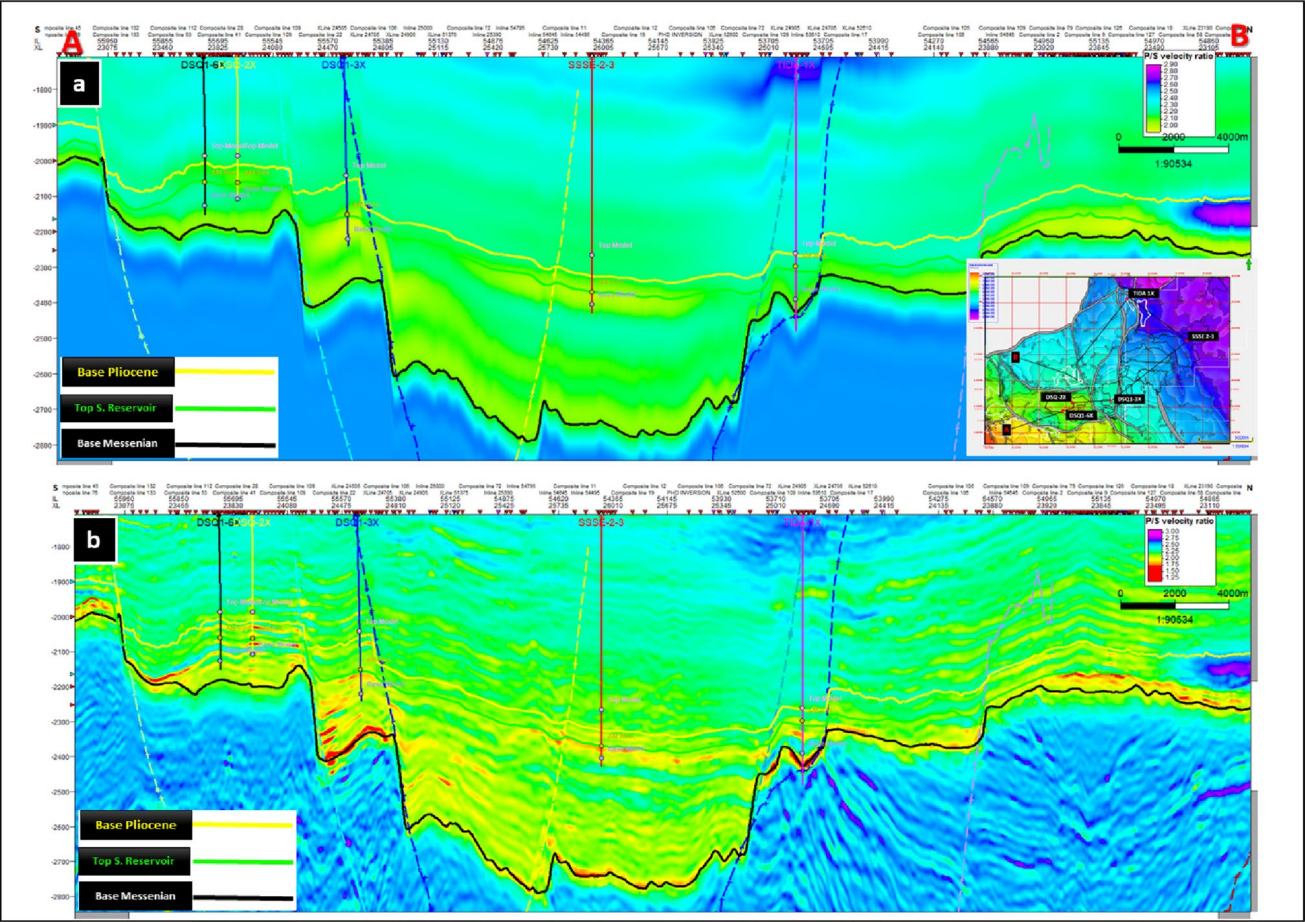
LFM (**a**) and inverted Vp/Vs (**b**) along the arbitrary line passing through the studied wells.

To constrain the inversion, two low-frequency models (LFMs) were constructed: one for **P-impedance** (Fig. 5a) and another for the **Vp/Vs ratio** (Fig. 6a). These LFMs were derived from well-log data and regional geological knowledge, ensuring that elastic property trends were consistent with the stratigraphic framework^23^. At the well locations, both models showed reliable impedance behavior and demonstrated vertically continuous Vp/Vs trends across the study area, confirming their geological validity.

Following inversion, the resulting **final elastic volumes** revealed substantial improvements in resolution. The **P-impedance inversion results** (Fig. 5b) displayed enhanced vertical and lateral continuity compared with its initial LFM, allowing clearer delineation of lithological variations. Similarly, the **Vp/Vs inversion results** (Fig. 6b) showed improved stratigraphic definition and highlighted reservoir compartmentalization, particularly within the Abu Madi sand channel systems^24,25^.

The refined inversion outputs provide a robust foundation for subsequent lithology prediction and facies classification. Figures 5 and 6 together present a direct comparison between the low-frequency models (Figs. 5a and 6a) and the inverted elastic volumes (Figs. 5b and 6b), clearly illustrating the marked improvements in vertical and lateral resolution^26,27^. These enhancements demonstrate how inversion transforms geologically constrained initial models into high-resolution elastic property volumes that capture subtle lithological variations and stratigraphic architecture, thereby increasing confidence in reservoir mapping^28^.

### Seismic inversion quality control

To evaluate and control the quality of inversion products, property datasets, and synthetic seismic responses, it is essential to compare these results against independent data sources such as well log measurements and established geological or geophysical models. The validation step confirms the inverted elastic-property volumes correlate strongly with the input well logs, thereby confirming the reliability of the inversion before proceeding with subsequent workflow stages. Rigorous quality assurance at this stage reduces uncertainty and enhances confidence in lithology prediction and reservoir characterization^29^.

The QC procedure typically involves cross-plotting inverted elastic attributes against well-log data, assessing the match between synthetic and observed seismic traces, and verifying consistency with prior geological models. Together, these checks provide evidence that the inversion outputs are both geologically reasonable and technically robust^21^.

**Fig. 7:**
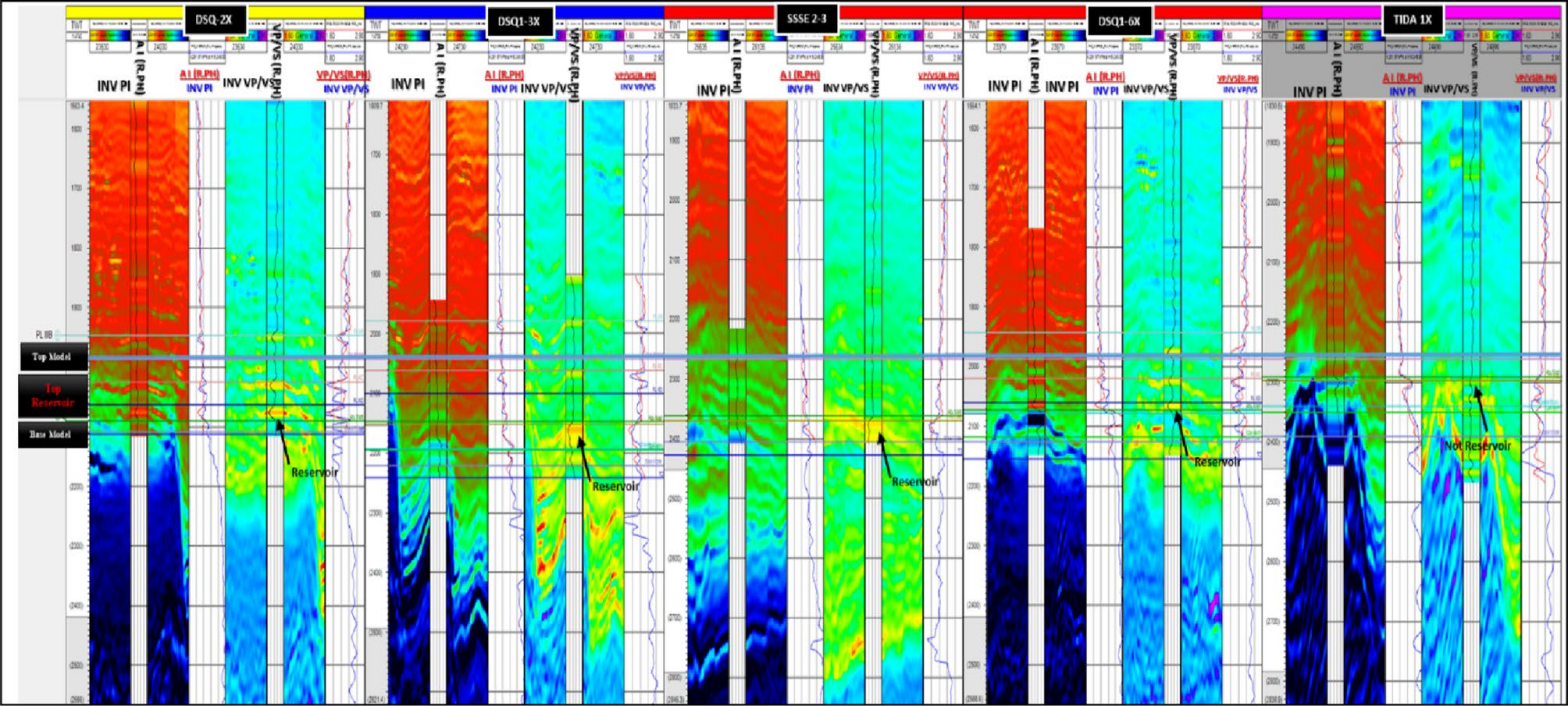
Integrated display of well data, prior models, and inversion results demonstrating an excellent match at the top reservoir sand in both the P-impedance and Vp/Vs inverted volumes.

Figure 7 illustrates the quality-control process applied to the inversion results across the studied wells. The panels display color-coded elastic-property volumes, such as P-impedance and Vp/Vs ratio, alongside corresponding well-log curves and synthetic seismic traces. Key reservoir intervals, including gas and oil-bearing zones, are clearly annotated to facilitate direct correlation.

The good agreement between the inverted elastic volumes and the well-log data confirms the geological validity of the inversion and supports its use for lithology prediction. This quality control step ensures the results align consistently with the measured data, thereby reducing uncertainty before lithology classification and subsequent reservoir modeling.

### Implemented workflow: stage 2

The second stage in the implemented workflow involves generating classification datasets from well logs through detailed petrophysical analysis and rock-physics-based facies definition. These calibrated relationships are then used to compute probability density functions (PDFs), which form the basis of the lithology classification model.

### Petrophysical data analysis

Petrophysical evaluation of well log data is a critical step in the identification and assessment of hydrocarbon-bearing zones. A detailed petrophysical evaluation provided by Suez Oil Company (SUCO) for the well logs of the studied five wells (DSQ-2X, DSQ1-3, DSQ1-6, Tida-1x, and SSSE2-3) that support rock physics modeling.

Table 1 presents the well pay summary for the Abu Madi Sand Reservoir after applying petrophysical cutoffs of 40% for clay content (Vcl), 8% for effective porosity, and 60% for water saturation (Sw). These thresholds were selected to isolate effective reservoir intervals with sufficient porosity and hydrocarbon saturation, while excluding clay-rich or water-dominated zones.

**Table 1:**
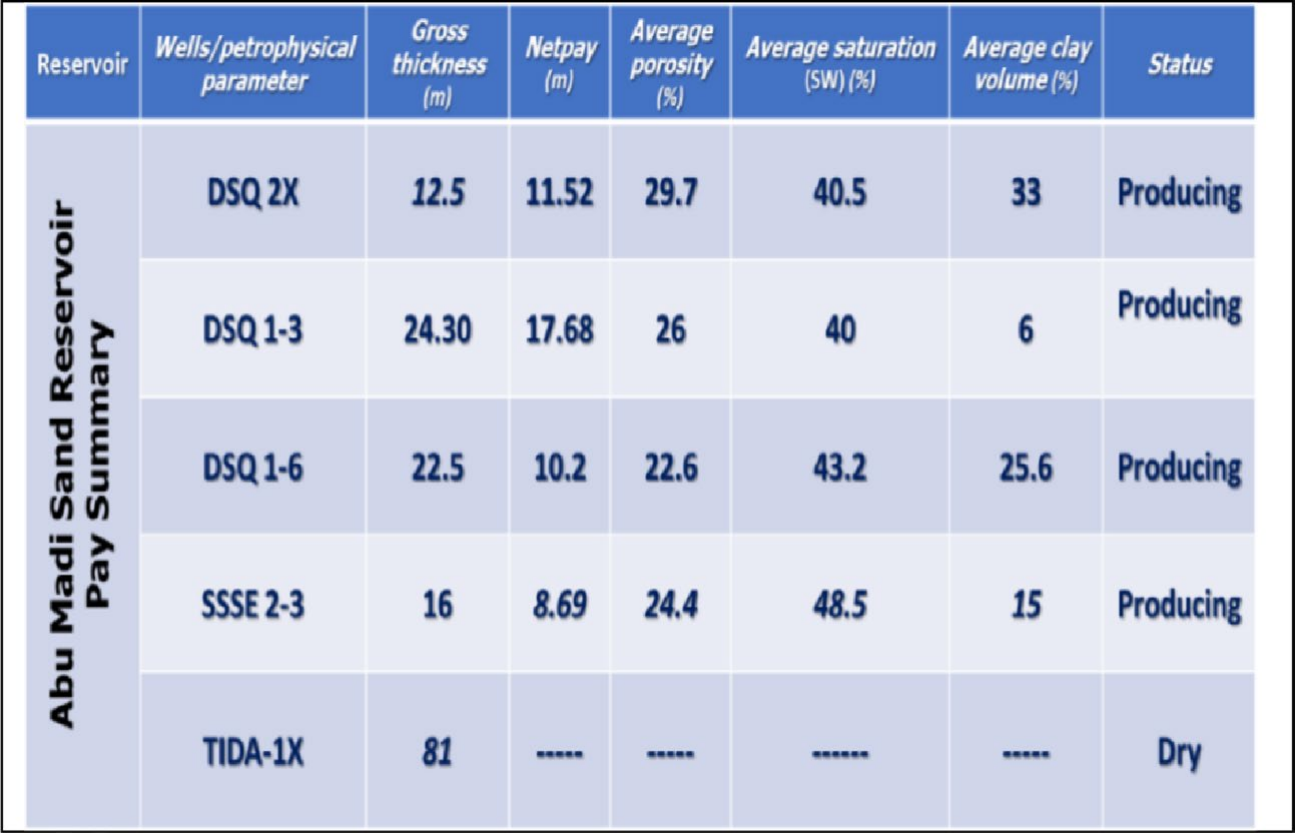
Studied wells pay zone summary.

Based on these criteria, four producing wells: DSQ 2X, DSQ 1–3, DSQ 1–6 and SSSE 2–3 are qualified as effective pay zones, while TIDA-1X well is excluded due to its clay-rich and/or water-dominated zones. This confirms a heterogeneous reservoir system with clean, high-quality sands in DSQ 1–3 and more clay-rich but still productive intervals in DSQ 2X, DSQ 1–6, and SSSE 2–3.

**Fig. 8:**
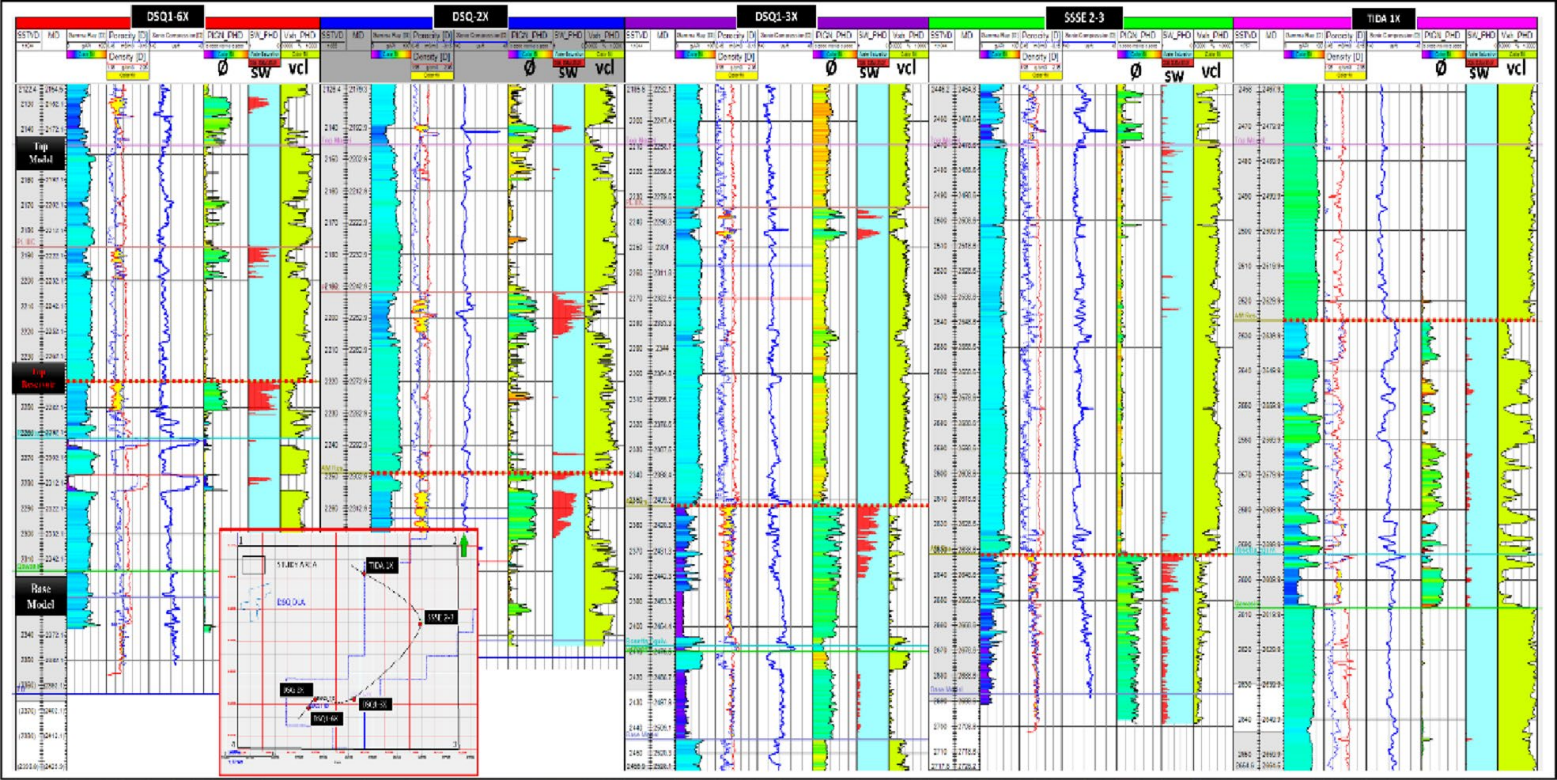
Estimated petrophysical parameters (Ø, Sw and Vcl) for Abu Madi sand reservoir.

These petrophysical parameters (Fig. 8) were subsequently used as a colored background during rock physics and class definition, providing a visual framework for analyzing lithological variations and fluid effects, and thereby enhancing the discrimination (separation) between hydrocarbon-bearing sands, brine sands, shale, and tight rocks.

### Rock physics and class definition

Rock physics is a fundamental discipline in geoscience that investigates the relationships between the physical properties of rocks and their geological characteristics, including mineral composition, pore structure, fluid content, and stress conditions^30^. It provides the theoretical and experimental framework that links measurable geophysical responses-particularly seismic velocities, density, and elastic moduli-to subsurface rock and reservoir properties^31^. By establishing quantitative models that describe how rocks respond to variations in lithology, porosity, saturation, and effective pressure, rock physics forms the critical bridge between geophysical observations and geological interpretation^32^.

Rock physics modeling was employed to quantitatively predict the relationships among elastic properties were generated to highlight lithological inconsistencies, discriminate pore-fluid types, and establish distinctions between hydrocarbon-bearing sands, brine sands, shale and tight formations. The generated elastic trends such as, acoustic impedance, shear impedance, and Vp/Vs ratios provide diagnostic signatures for facies classification and fluid detection^33–35^.

In petroleum geophysics, rock physics plays a central role in translating seismic data into meaningful petrophysical parameters, thereby enabling improved reservoir characterization, lithology discrimination, and fluid identification^21^.

Rock physics concepts support key quantitative interpretation workflows such as seismic inversion, amplitude variation with angle (AVA) analysis, and elastic attribute analysis^30,36^.

Consequently, rock physics represents a basis of integrated geophysical-geological analysis, providing physically consistent models that reduce uncertainty in subsurface property prediction and decision-making.

These established relationships between elastic properties enabled the identification of diagnostic clusters and trends in elastic property space. These clusters provided robust facies and fluid indicators, allowing for the discrimination of hydrocarbon-bearing sands, brine-saturated sands, shale, and tight formations^37,38^.

This information was subsequently integrated into the seismic inversion workflow, serving as calibration points that enhanced the reliability of inversion outputs.

As a result, lithology predictions across the reservoir were significantly improved, reducing interpretation risk and providing a more accurate representation of the Messenian depositional system. This information was subsequently used to calibrate the seismic inversion results, resulting in improved lithology predictions throughout the reservoir.

Despite the availability of high-quality seismic data and reliable well control, accurate subsurface interpretation requires a quantitative understanding of the relationship between geology and seismic response. Traditional qualitative seismic interpretation is mainly concerned with structural and stratigraphic interpretation utilizing reflection data. In contrast, quantitative seismic interpretation techniques have been developed with the aim of deriving information concerning rock and fluid properties from the seismic amplitude waves. Within this context, rock physics plays an important role by linking elastic parameters to lithology and pore-fluid content, thereby reducing interpretation risk^21^.

In this study, detailed multi-attribute cross-plots were generated using available well logs to characterize and discriminate lithologies across the study area. The primary inputs for the rock-physics analysis included compressional-wave velocity (Vp), shear-wave velocity (Vs), and density logs. Integration of these elastic parameters enabled the creation of cross-plots that effectively distinguish between key lithological facies such as gas-bearing sands, wet sands, shales, and anhydrites^25,27^.

These relationships enabled the identification of diagnostic clusters and trends in elastic property space. This information was subsequently used to calibrate the seismic inversion results, resulting in improved lithology predictions throughout the reservoir.

**Fig. 9:**
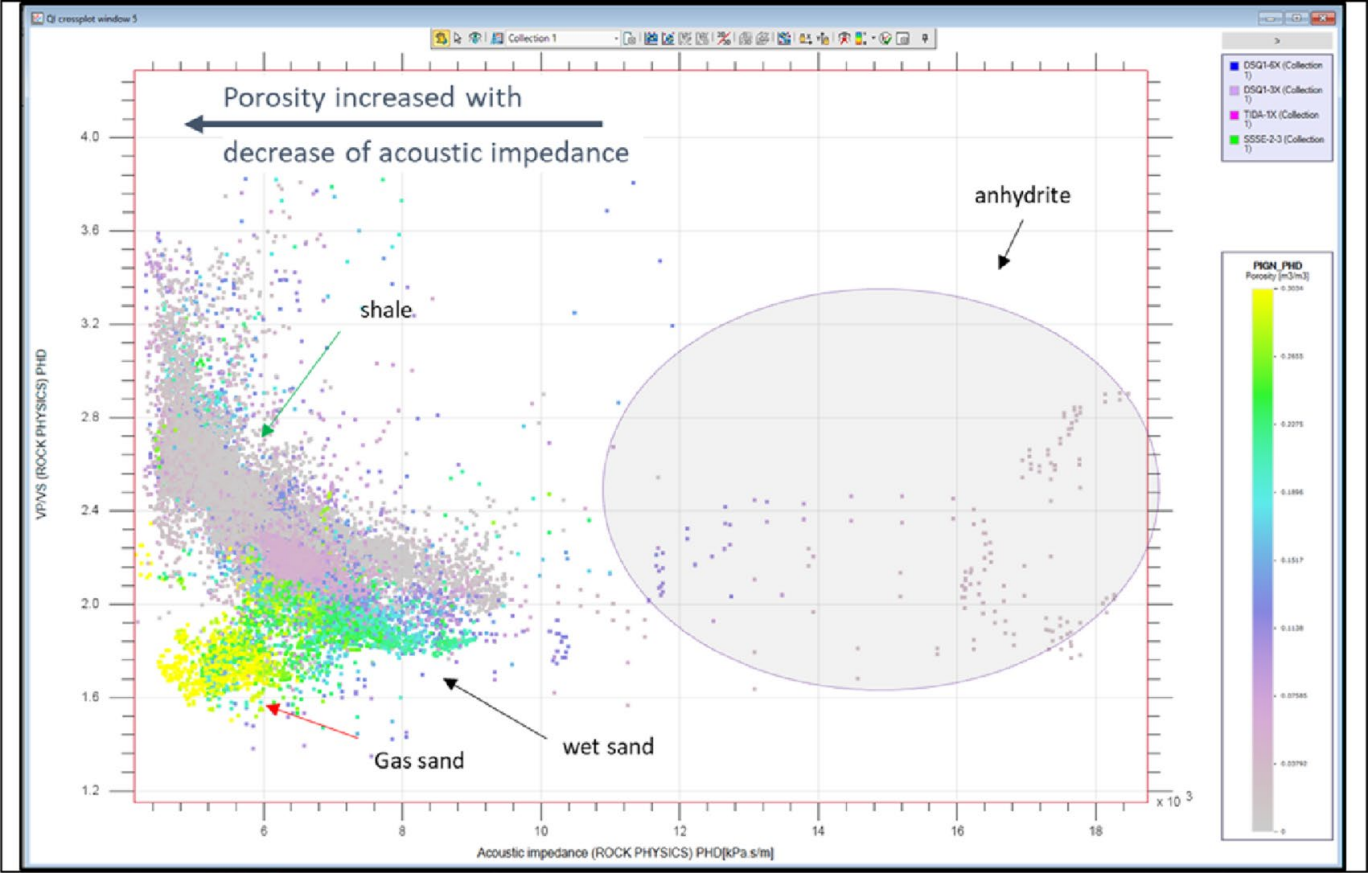
A crossplot of Vp/Vs ratio versus acoustic impedance, color-coded by porosity, revealing distinct lithological and fluid-related trends.

Figure 9 presents a crossplot of Vp/Vs ratio versus acoustic impedance, color-coded by porosity, revealing distinct lithological and fluid-related trends. As acoustic impedance decreases, porosity increases, indicating a transition from dense, compact rocks to more porous formations. Anhydrite occupies the high-impedance, high-Vp/Vs domain, reflecting its stiff, non-porous nature. Shales plot in the upper-middle region with moderate impedance and elevated Vp/Vs values, consistent with their clay-rich composition and moderate porosity. Wet sands exhibit intermediate impedance and Vp/Vs characteristics indicative of water-saturated reservoir zones. In contrast, gas-bearing sands display the lowest acoustic impedance and Vp/Vs values, making them readily distinguishable in elastic-parameter space.

**Fig. 10:**
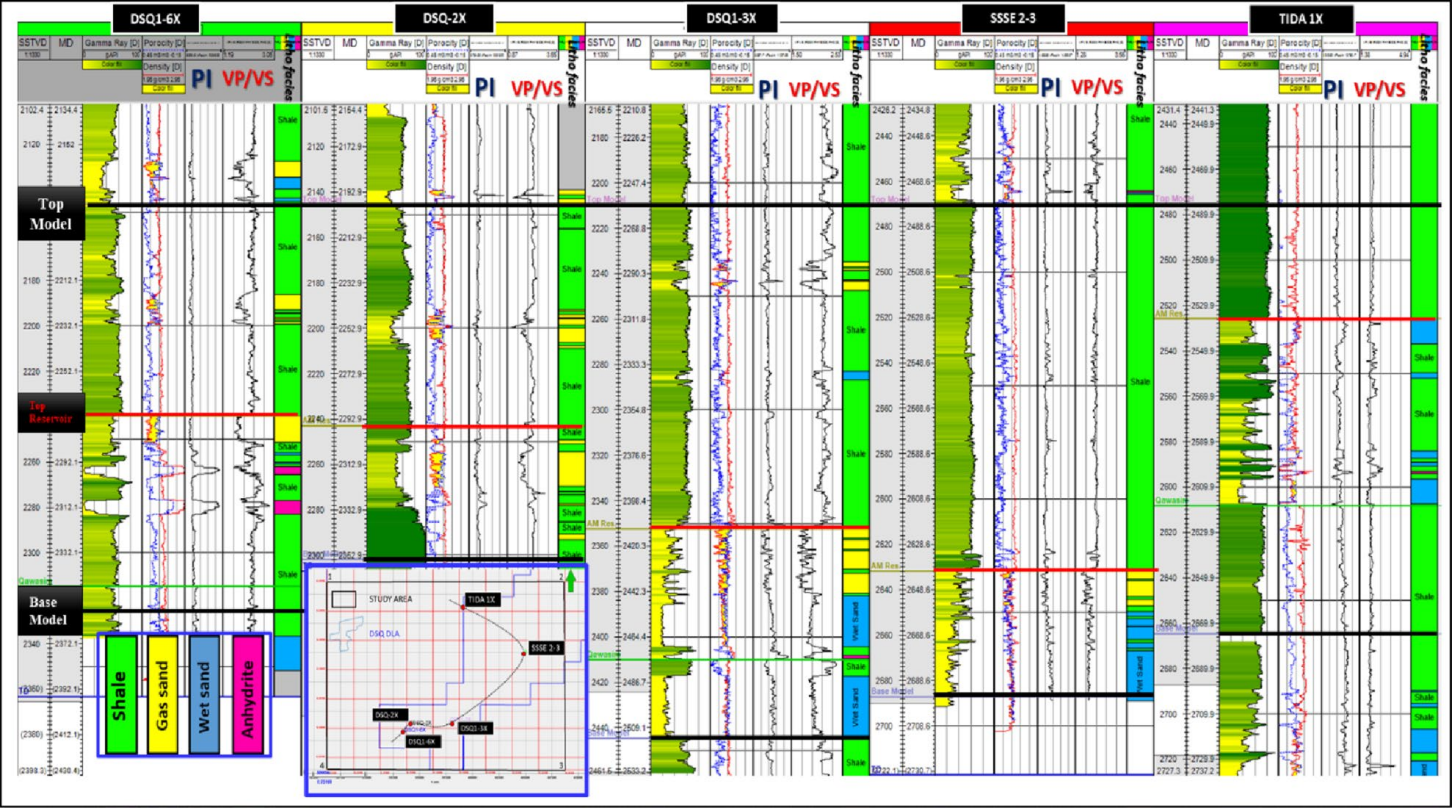
Well-correlation panel for the studied wells illustrating the lithology classification log derived from the rock-physics analysis.

Therefore, lithology classification log was established for all studied wells and showed strong correlation with measured well data and reveals a good fluid, lithology discrimination such as gas-bearing sands, wet sands, shales, and tight formation (anhydrites), as shown in Fig. 10.

### Probability density functions (PDFs) (litho classification model)

The **lithology classification log** (Fig. 10), derived from rock physics relationships represents the main input for the lithology analysis process. This workflow enables the creation of probability density functions (PDFs), which quantify the likelihood of different lithological facies across the reservoir.

The PDFs illustrate the frequency distribution of samples relevant to each lithological class and represent the variability in formation properties. The log properties were subsequently upscaled to seismic properties, with the PDFs aligned to the expected seismic inversion accuracy, ensuring consistency between well-based rock physics and seismic-derived volumes by using a fully Bayesian approach^2,3,7^.

**Fig. 11:**
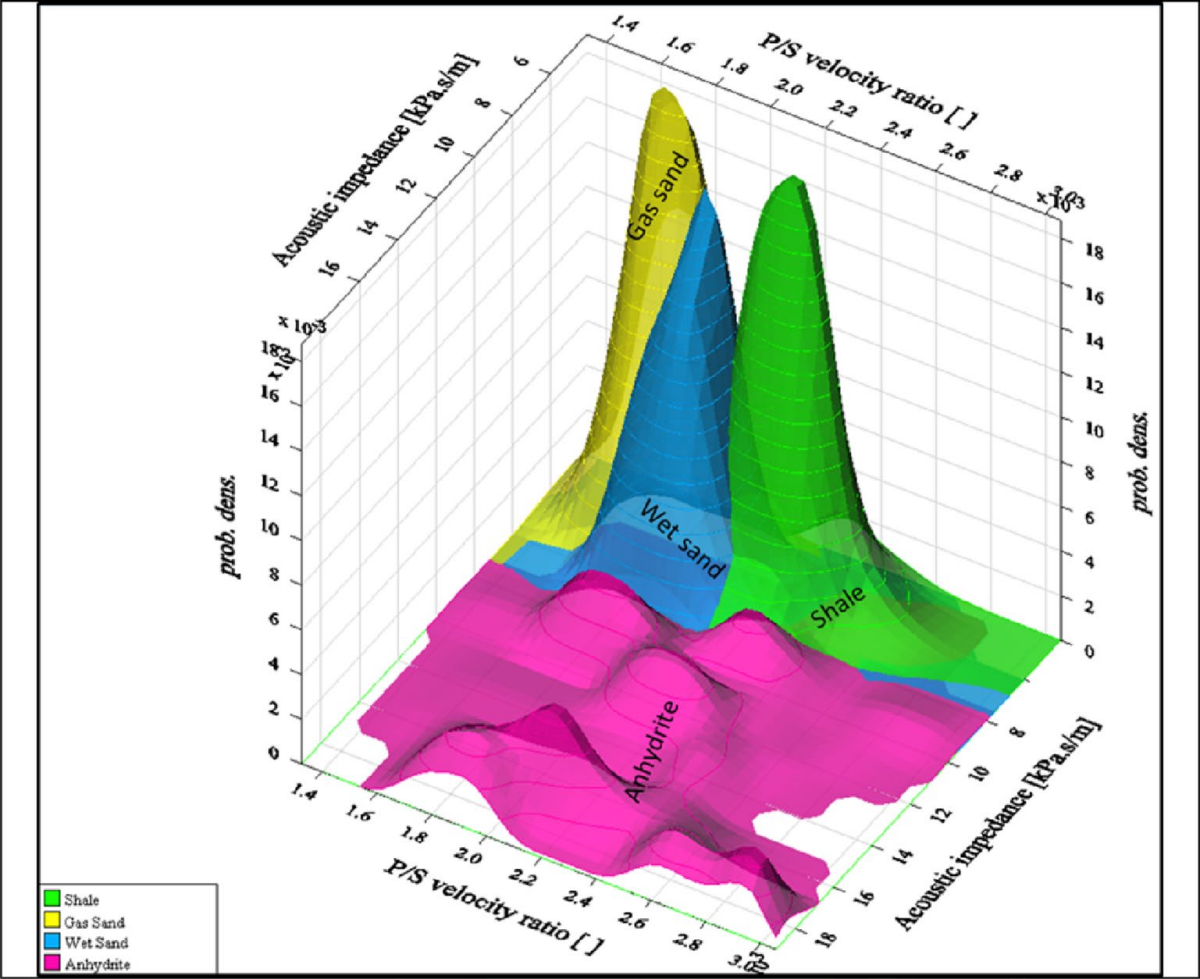
3D cross-plot illustrating the probability distribution functions (PDFs) of the discriminated lithologies, including shale, wet sand, gas sand, and anhydrite.

**The 3D plot clearly** illustrates how P-impedance and Vp/Vs ratio together enhance facies separation and confirms that **gas sand** is the most promising reservoir facies, with a **distinct** elastic signature in both P-impedance and Vp/Vs ratio. Shale and Wet Sand are clearly separated, supporting reliable classification. Anhydrite is non-reservoir but structurally distinct. This visualization is ideal for facies classification, reservoir targeting, and synthetic gather calibration, as illustrated in Fig. 11 (yellow color is the gas sand, blue color is the wet sand, green color is the shale and magenta color is the anhydrite probability density functions.

### Confusion matrix and statistics QC

**Fig. 12:**
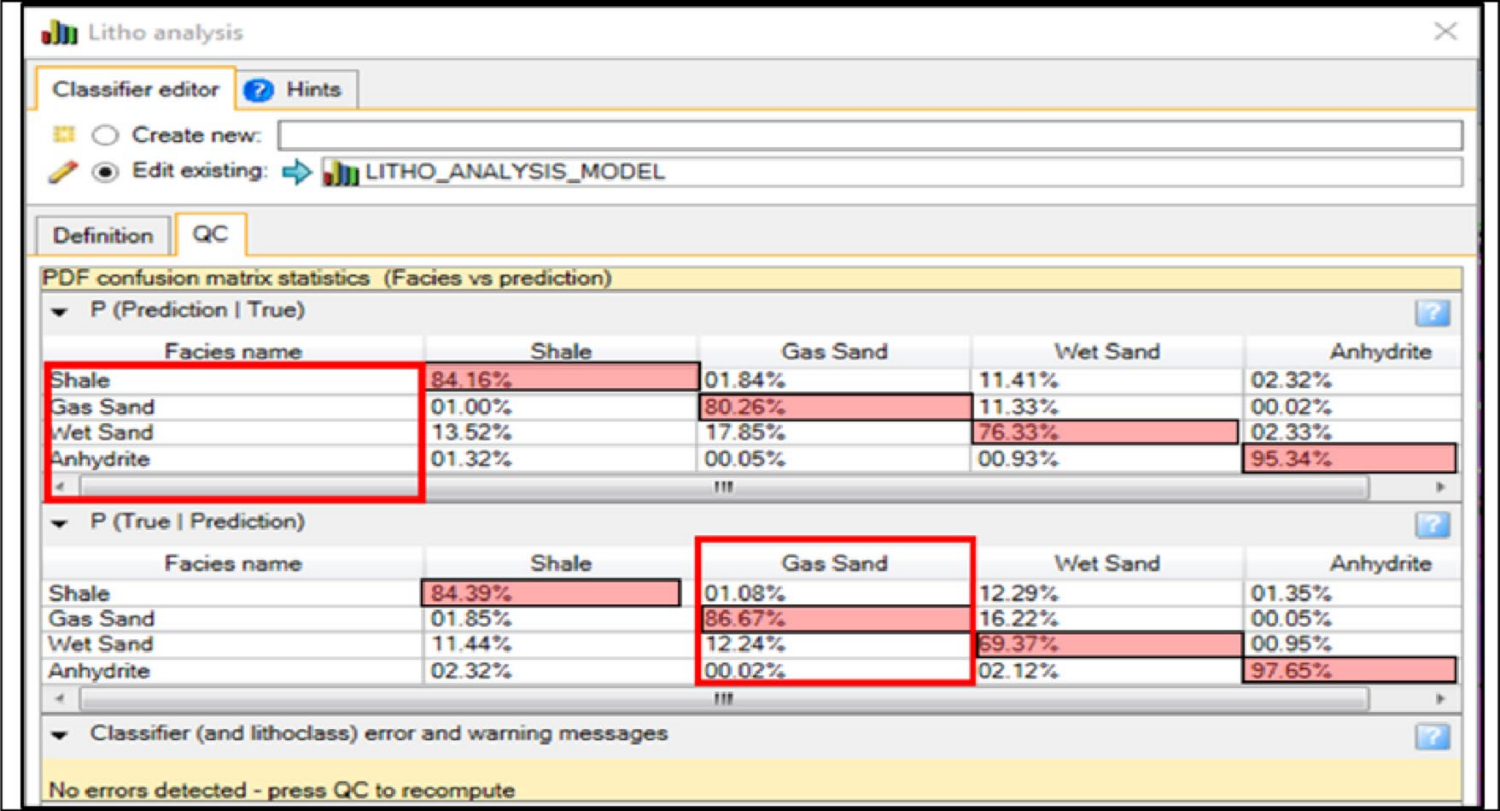
Confusion matrix illustrating the comparison between predicted lithology classes and their corresponding facies-based probability density functions (PDFs).

The confusion matrix allows the user to analyze the results and performance of a specific algorithm, which classifies data by Bayesian classification^7^. The QC contains two Tables (P (Prediction |True) and P (True | Prediction)), shown in Fig. 12. The left-hand side of the tables (the rows) contains the classes that are defined, while the top of the tables (the columns) contains the predicted classes and get high-confidence classification results, the values along the diagonal of this matrix should be as large as possible where:

- **P (Prediction |True) table** shows the probability of a classification occurring given the true class (Prediction I True).
- 1.84% of the samples that belong to the shale class were predicted as gas sand.
- 80.26% of the samples that belong to the gas sand class were predicted as gas sand.
- 17.85% of the samples that belong to the wet sand class were predicted as gas sand.
- 00.05% of the samples that belong to the anhydrite class were predicted as gas sand.
- **P (True | Prediction) table** shows the probability of a sample belonging to a particular class, given the predicted class (True I Prediction).
- The samples that belong to the shale class have a probability of 1.08% of being classified as a gas sand class.
- The samples that belong to the shale class have a probability of 86.6% of being classified as a gas sand class.
- The samples that belong to the wet sand class have a probability of 12.24% of being classified as a gas sand class.
- The samples that belong to the anhydrite class have a probability of 00.02% of being classified as a gas sand class.

### Lithology prediction and probability cubes

The lithology prediction process, is the integration of the probability density functions (PDFs) derived from rock physics modeling and the inverted elastic attribute volumes generated by seismic inversion (P-impedance and Vp/Vs) serve as the primary inputs for this analysis to produce lithology and fluid prediction volumes along with their associated uncertainties.

This probabilistic approach allows for the quantification of facies distribution across the reservoir; highlighting zones of gas sand, wet sand, shale, and anhydrite. The resulting prediction volumes provide a robust framework for reservoir characterization, ensuring that lithology interpretation is consistent with both well log data and seismic inversion accuracy^2,3,39^.

**Fig. 13:**
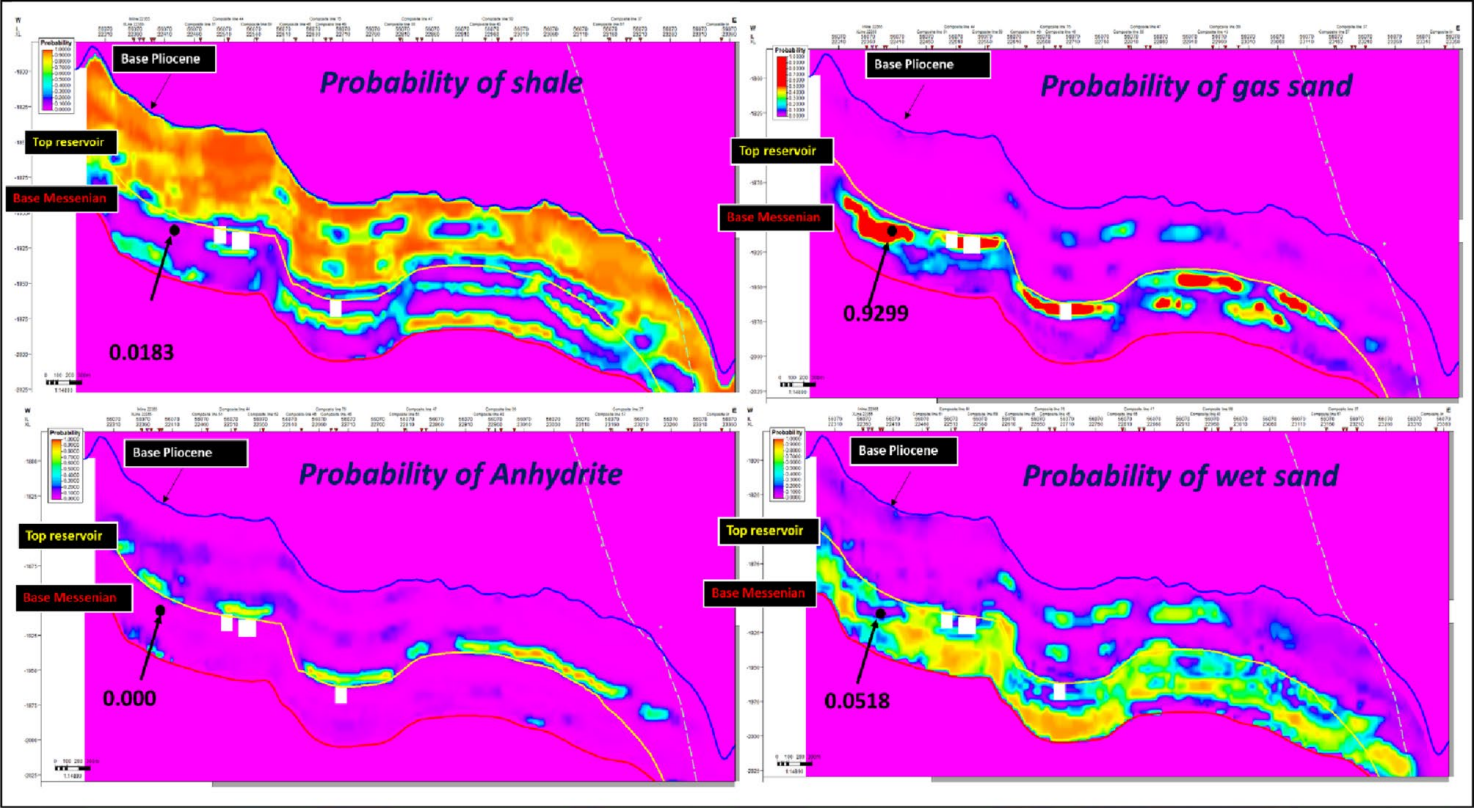
Facies-probability sections showing high gas-sand probability, negligible anhydrite, and low shale and wet-sand probabilities, tied to key stratigraphic markers. With all probabilities summing to 1.0.

Figure 13 illustrate Facies-probability vertical sections derived from the property cubes, showing high gas-sand probability (0.9299), negligible anhydrite (0.0000), and low shale (0.0183) and wet-sand (0.0518) probabilities, with all probabilities summing to 1.0 at a selected point (black dot). These probability sections are tied to key stratigraphic markers including the Base Pliocene, Top Reservoir, and Base Messinian and are calibrated to the resolution of the seismic inversion results.

## Results

The output lithology probability cubes, demonstrate a strong correlation of high confidence with well data.

The close match between predicted facies and measured elastic properties confirms the geological realism of the inversion and classification outputs. Furthermore, the confusion matrix analysis supports the robustness of the probabilistic approach, with gas sand facies showing the highest prediction accuracy.

This correlation validates the reliability of the probabilistic classification workflow and confirms the presence of compartmentalized gas sand channels within the Abu Madi Formation.

The 3D probability volumes highlights a new gas-bearing areas compared to that was previously interpreted as a tight lithology impact (high-amplitude anomalies), thereby reducing uncertainty in reservoir characterization.

Overall, the visualization and QC process demonstrates that the integrated workflow not only improves facies discrimination but also updates the understanding of gas distribution in the study area, providing a reliable foundation for reservoir development planning.

## Discussion

### Fluid probabilistic identification

**Fig. 14:**
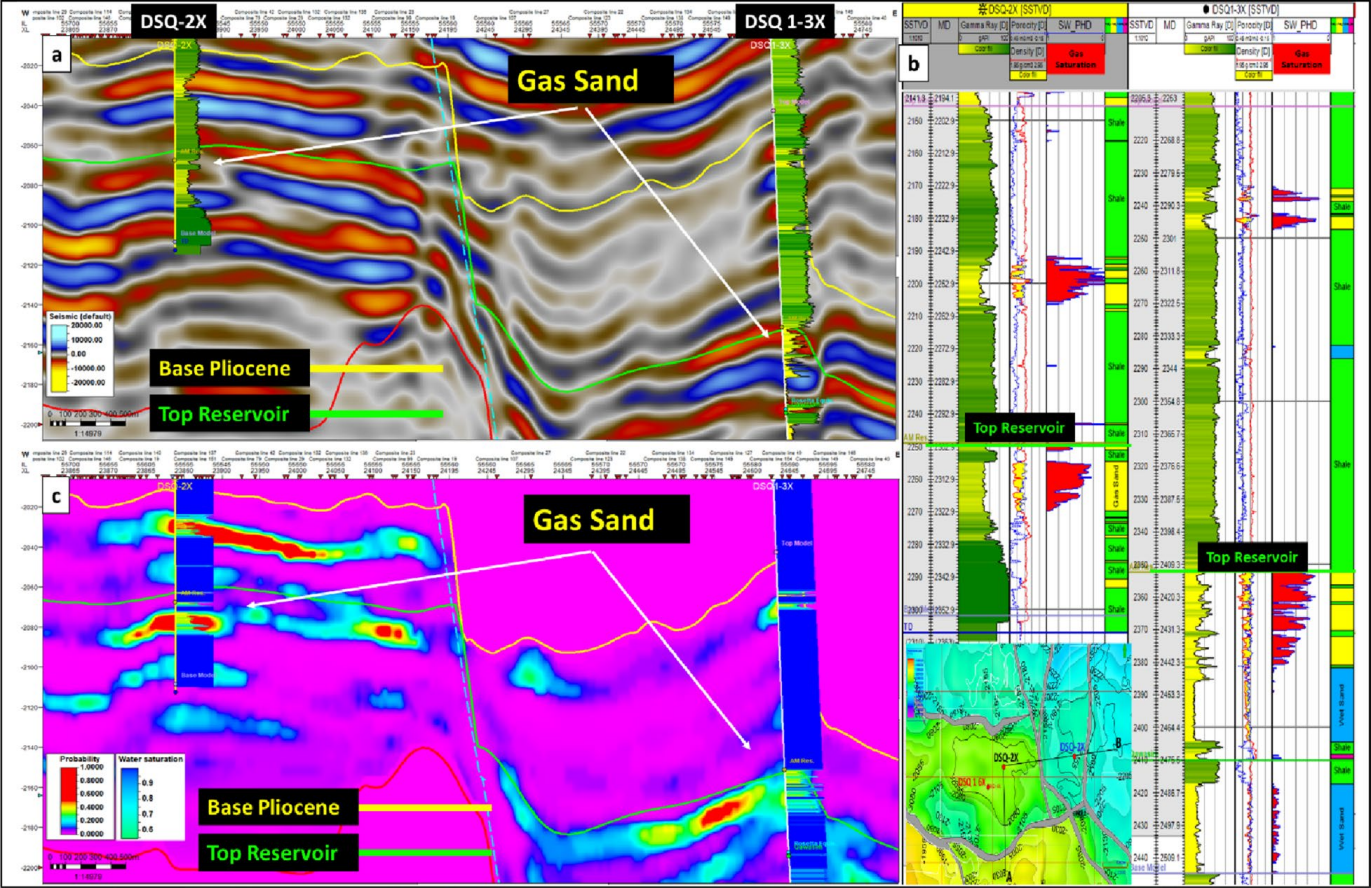
Conventional 3D seismic line (**a**), Composite well logs of DSQ-2X and DSQ1-3X (**b**) and Gas-sand probability section (**c**).

Figure 14 illustrates that the probabilistic approach effectively integrates seismic and well log data, yielding a reliable discrimination of fluid facies and enhancing reservoir evaluation.

Figure 14a: Shows the conventional 3D seismic line overlaid by GR logs between two productive wells DSQ-2X and DSQ-1-3X with two interpreted seismic horizons (Base Pliocene and Top reservoir) highlighting the gas bearing sand reservoir represented by low impedance negative amplitude (Red color).

Figure 14b: highlight the water saturation curve (Blue curve) flagged with gas saturation (red color fill) against the cross over neutron density log that proved the gas bearing sand reservoir.

Figure 14c: represent the derived gas sand probability section extracted from the gas-sand probability cube, overlaid with water saturation logs of DSQ-2X and DSQ-1-3X wells, demonstrates strong agreement between probabilistic outputs and petrophysical measurements, where zones of high gas sand probability coincide with intervals of low water saturation, confirming the reliability of the Bayesian probabilistic workflow in discriminating gas bearing sands from surrounding tight formations.

### Lithology probabilistic identification

The overlap between the probability cube and the well logs confirms a high level of confidence in lithology prediction, validating the reliability of the probabilistic workflow in discriminating gas bearing sands from surrounding tight formations and resolving ambiguities caused by thin, laterally discontinuous tight lithologies.

**Fig. 15:**
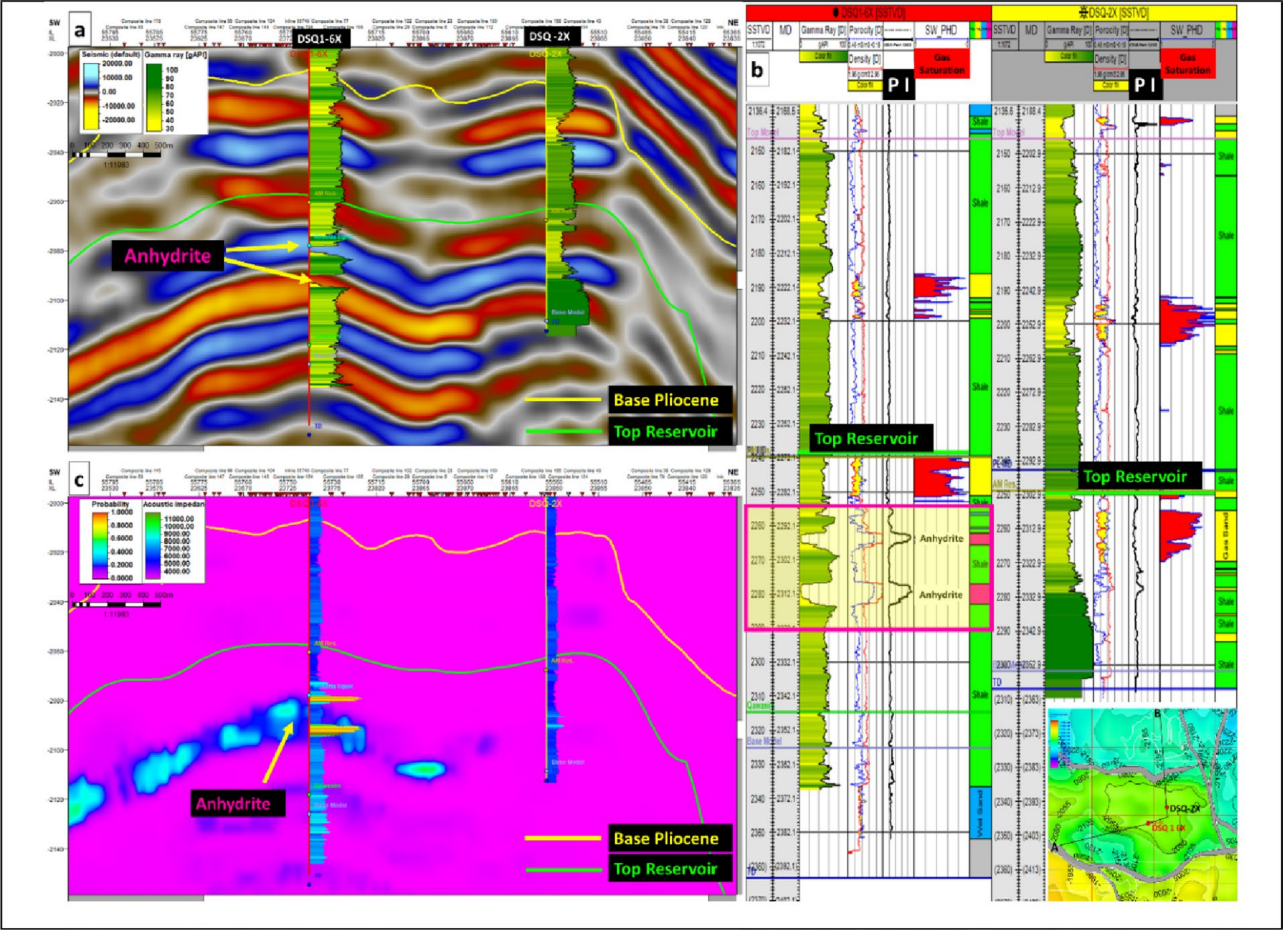
Conventional 3D seismic line (**a**), Composite well logs of DSQ1-6X and DSQ-2X (**b**) and anhydrite (tight lithology) probability section (**c**).

Figure 15 illustrates Facies probability section extracted from the generated tight lithology (Anhydrite) probability cube, overlaid with P-impedance logs from wells DSQ1-6X and DSQ-2X. The section highlights zones of tight lithology in DSQ1-6X, where two distinct anhydrite layers of relative thickness 3–6 m are penetrated.

Figure 15a: Shows the conventional 3D seismic line overlaid by GR logs between two productive wells DSQ1-6X and DSQ-2X with two interpreted seismic horizons (Base Pliocene and Top reservoir), highlighting the thin anhydrite bed exert a significant impact on the contrast between peak and trough impedances, producing amplitude “booming” effects that complicate seismic interpretation. The conventional seismic reflection data suggest a lateral extension of anhydrite beds recorded in DSQ1-6X toward DSQ-2X represented by high impedance positive amplitude (Blue color), despite of DSQ-2X well not contain any anhydrite bed Fig. 15b, that consider the main challenge while seismic interpretation.

Figure 15b: highlight the anhydrite beds recorded in DSQ1-6X well (Yellow colored box), while DSQ-2X well not penetrated any tight beds.

Figure 15c: represent the derived facies-probability section extracted from the tight lithology (Anhydrite) probability cube, overlaid with P-Impedance logs of DSQ1-6X and DSQ-2X wells, the probabilistic cube reveals thin anhydrite layers at DSQ-1-6X and their limitation of extension toward DSQ-1-2X, that coincide with intervals of high P-Impedance logs.

### Prospects and gas sand outlines

**Fig. 16:**
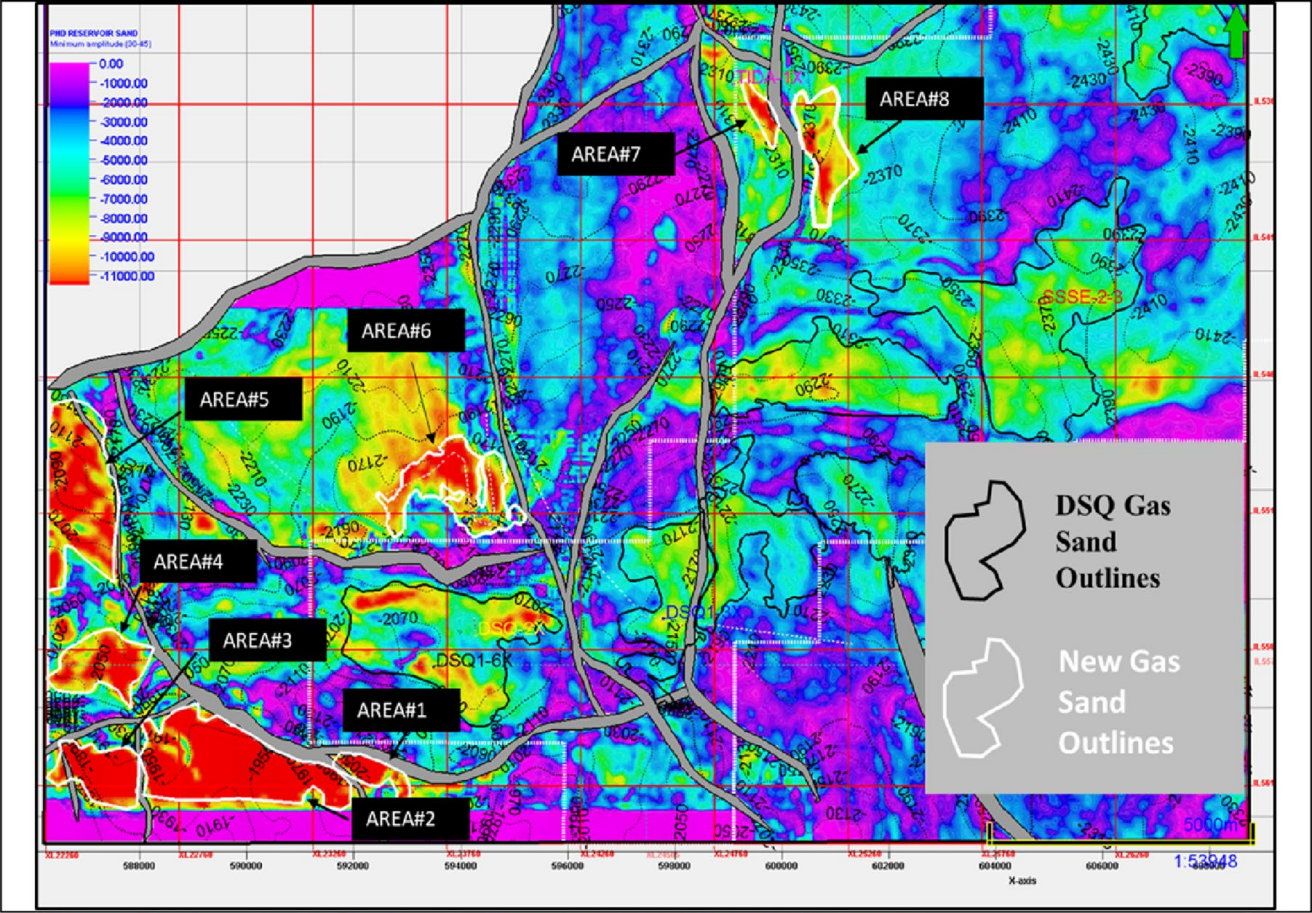
Minimum amplitude map on top reservoir shows Gas-sand distribution in the Desouq Gas Field (black polygons), refined using probabilistic lithology cubes. The workflow reduces uncertainty from anhydrite-related amplitudes and identifies eight additional gas-sand zones (white polygons), improving reservoir delineation and interpretation confidence.

Figure 16 illustrates the agreed and discovered outlines of gas sand distribution in the Desouq Gas Field, annotated by black polygons^40^. These reservoir bodies were initially discovered based on amplitude maps and conventional inversion elastic properties. These outline is highlighted based on conservative interpretations carried uncertainty due to the possible presence of anhydrite facies, which attributed to misleading amplitude responses and complicate reservoir delineation.

The integration of probabilistic cubes (lithology and fluids), the interpretation vision becomes broader and more reliable, thus the probabilistic workflow enables improved tracking of anhydrite facies, thereby minimizing the risk of misclassification and reducing uncertainty in reservoir mapping. That resulted in adding eight new gas sand outlines, annotated by white polygons. These zones are characterized by very strong amplitude anomalies (booming amplitudes), which are now confidently attributed to gas sand facies rather than thin anhydrite layers.

## Conclusions

This study demonstrates the effectiveness of integrating advanced seismic inversion techniques with rock-physics modeling and probabilistic lithology classification to enhance reservoir characterization in the onshore Nile Delta. The 3D probability lithology cubes developed in Petrel™ by using QI module.

Pre-stack simultaneous inversion generated key elastic-property volumes (P-impedance and Vp/Vs ratio), which were calibrated against well-log measurements and applied to achieve accurate facies discrimination. the probabilistic lithology classification model,, produced probability density functions (PDFs) that capture reservoir variability and support reliable lithology prediction across the seismic volume.

PDFs validated by the Confusion matrix analysis. Gas sand facies show the highest prediction accuracy (86.6%), while shale and wet sand exhibit minor overlap (1.08% and 12.24% respectively). Anhydrite misclassification is negligible (0.02%).

The resulting 3D lithology cubes reveal compartmentalized gas‑sand channels within the Abu Madi Formation of the Desouq Field and identified eight additional gas‑charged zones in the southwest sector of the Desouq Field, previously obscured in conventional interpretations. This cubes were validated by the petrophysical data of the studied wells.

Earlier interpretations based on amplitude maps and deterministic inversion often misclassified tight lithologies, particularly thin anhydrite layers that occur above or below the sand reservoirs. These high impedance contrasts generate misleading amplitude responses that mimic gas sands. By contrast, the probabilistic workflow improves facies discrimination, reduces uncertainty, and aligns with global applications of Bayesian classification in complex depositional settings, ultimately increasing confidence in reservoir mapping.

Despite the improved accuracy of the Bayesian classification, some uncertainties remain. The primary challenge is the overlap between wet sand and gas sand, which share similar elastic properties and therefore lead to misclassification.

Similarly, deterministic seismic inversion is inherently limited by the vertical resolution of the input seismic data; the inversion results cannot exceed this resolution. Thin anhydrite layers that fall below the vertical seismic resolution also remain difficult to identify reliably, introducing further ambiguity. These limitations stem both from the intrinsic physical properties of the rocks and from seismic data resolution constraints. Additional factors such as seismic noise, multiples, and acquisition footprints further degrade accuracy and contribute to uncertainty.

Together, these issues highlight the Compromises between deterministic and probabilistic approaches: while Bayesian classification improves facies discrimination and reduces uncertainty, challenges related to elastic property overlap, thin bed resolution, and seismic noise remain intrinsic to the data and must be carefully considered in reservoir characterization workflows.

Future research should focus on incorporating multi-attribute classification, refining rock physics constraints, and extending the probabilistic workflow to other Nile Delta concessions. Such efforts will further reduce uncertainty, enhance facies discrimination, and broaden the applicability of this methodology to complex depositional settings worldwide.

## Data Availability

The raw data used in the current study remains confidential to the Egyptian General Petroleum Corporation (EGPC) and used with permission and is not publicly available. Data was available to authors with permission from EGPC (the Egyptian General Petroleum Corporation), EGPC email: [EGPC@egpc.com.eg](mailto: EGPC@egpc.com.eg).
